# Sex differences in autonomic recovery following repeated sinusoidal resistance exercise

**DOI:** 10.14814/phy2.15269

**Published:** 2022-04-24

**Authors:** Joseph Carere, Joel S. Burma, Kailey T. Newel, Courtney M. Kennedy, Jonathan D. Smirl

**Affiliations:** ^1^ 2129 Cerebrovascular Concussion Lab Faculty of Kinesiology University of Calgary Alberta Canada; ^2^ 2129 Sport Injury Prevention Research Centre Faculty of Kinesiology University of Calgary Calgary Alberta Canada; ^3^ 2129 Hotchkiss Brain Institute University of Calgary Calgary Alberta Canada; ^4^ 2129 Integrated Concussion Research Program University of Calgary Calgary AB Canada; ^5^ Alberta Children’s Hospital Research Institute University of Calgary Calgary Alberta Canada; ^6^ 2129 Human Performance Laboratory Faculty of Kinesiology University of Calgary Calgary Alberta Canada; ^7^ Libin Cardiovascular Institute of Alberta University of Calgary Alberta Canada; ^8^ Faculty of Health and Exercise Science University of British Columbia Kelowna British Columbia Canada

**Keywords:** autonomic nervous system, baroreceptor sensitivity, heart rate variability, resistance exercise, squat‐stand maneuvers

## Abstract

A simple bodyweight squat is sufficient to cause substantial stress on the autonomic nervous system (ANS) via ~30–50 mmHg blood pressure (BP) oscillations. However, it is unknown to the extent of the ANS is impacted during and immediately following bodyweight and resistance squat‐stand maneuvers (SSM) while considering chromosomal sex. Thirteen females and twelve males performed four, 5‐minute bouts of squat‐stand maneuvers (SSM); two at 0.05 Hz (10‐second squat/10‐second stand) and two at 0.10 Hz (5‐s squat/5‐s stand). The SSM were performed using bodyweight resistance and additional external resistance (~20% of bodyweight). Five‐minutes of quiet‐sitting and quiet‐standing were completed immediately following both bodyweight and resistance squats. Heart rate variability (HRV) and baroreceptor sensitivity metrics were extracted from beat‐to‐beat electrocardiography and systemic BP recordings. Repeated measure Analysis of Variance with generalized eta‐squared effect sizes assessed differences between SSM task type and chromosomal sex on ANS metrics. Despite added resistance eliciting greater elevations in blood pressure, no differences in ANS function were noted during competition and recovery between SSM tasks (all *p* > 0.050; negligible/small effect sizes). During recovery, females had an elevated heart rate (*p* = 0.017; small effect size), greater time‐domain HRV measures (*p* < 0.047; small effect size), greater high‐frequency domain HRV measures (*p* = 0.002; moderate effect size), and reduced low‐frequency domain HRV measures (*p* = 0.002; moderate effect size). A healthy ANS can modulate repetitive cardiovascular stressors via squat‐stand maneuvers in a harmonious manner irrespective of added low‐level resistance. Females were more parasympathetically driven following low‐level resistance exercise/stress, which may be a cardioprotective trait.

## Graphical abstract

Cardiovascular stressors accompanying light resistance exercise did not elicit changes to autonomic modulation and recovery compared to bodyweight exercise. A sex effect was discovered, as females displayed stronger vagal tone during recovery from light resistance exercise. Combined with findings from previous literature, the present results suggest a blood pressure threshold may exist in which females no longer display stronger vagal recovery. Analysis of automimic function surrounding resistance exercise may hold future implications for concussion recovery.

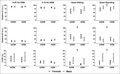

## 1 INTRODUCTION

The autonomic nervous system (ANS) is a key regulator of involuntary physiological processes, such as cardiac activity and systemic blood pressure (BP) regulation (Shaffer & Ginsberg, 2017). At rest, the parasympathetic (PNS) and sympathetic (SNS) branches of the nervous system work to counterbalance physiological processes in order to maintain homeostasis (Shaffer & Ginsberg, 2017). Previous research has elucidated the utility of assessing ANS function in both healthy and clinical populations through heart rate variability (HRV; cardiac activity) and baroreceptor sensitivity (BRS) metrics (Acharya et al., 2006; La Rovere et al., 2008).

With the onset of exercise, a concurrent response of parasympathetic withdrawal and elevated sympathetic activity occurs, resulting in increased heart rate (HR) to meet metabolic demands (Fontolliet et al., 2018; Freeman et al., 2006). However, this process is partially dependent on the mode and intensity of exercise completed, as cardiovascular responses to resistance exercise differ greatly from aerobic exercise (Sharman & Lagerche, 2015). For example, during a maximal treadmill test, normative systolic/diastolic BP have shown to rise to ~180/70 mmHg (mean arterial pressure [MAP] ~105 mmHg) and ~160/70 mmHg (MAP ~100 mmHg) for males and females, respectively (Sharman & Lagerche, 2015). During maximal resistance exercise (e.g., squats), MAP can be augmented upwards of 300–350 mmHg, creating a substantially different stimulus for the body and brain to buffer (MacDougall et al., 1985; Palatini et al., 1989). These extreme BP swings elicited by resistance exercise require augmented regulatory control mechanisms in order to maintain homeostasis through autonomic (e.g., baroreflex) and other body systems (e.g., endothelial) (Davis et al., 2003; Di Francescomarino et al., 2009).

Nevertheless, as reviewed in Goldsmith, Bloomfield, and Rosenwinkel (2000), Hautala, Kiviniemi, and Tulppo (Hautala et al., 2009), and Seals et al (Seals et al., 2001), the impact of aerobic exercise on cardiac activity and BRS has widely been assessed both during and following aerobic exercise. However, fewer studies have investigated the role resistance exercise has on acute ANS activity (Heffernan et al., 2006; Isidoro et al., 2017; Mayo et al., 2016; Mayo et al., 2016). This may in part be attributable to the time constraints required to obtain valid and reliable ANS data (5‐min) (Burma, Graver, et al., 2021), where commonly prescribed forms of resistance exercise are not sustainable for greater than 15 repetitions (~30‐seconds). However, squat‐stand maneuvers (SSM) have been widely used throughout the cerebral autoregulatory literature, which is a form of low‐level resistance exercise that can be completed for 5‐minutes or greater (Smirl et al., 2015) These are known to elicit ephemeral BP fluctuations of ~30–50 mmHg with the loading and unloading of each individual squat (Smirl et al., 2015), which activates the baroreflex to respond to the cardiovascular stressor (Zhang et al., 2009). Nevertheless, only bodyweight SSM has been used within the literature (Smirl et al., 2015; Zhang et al., 2009). Hence the cardiovascular changes that occur with added resistance during these maneuvers remains unknown.

Previous literature has also stressed the underrepresentation of female participants as a large limitation in cardiovascular research, as reporting differences based on chromosomal sex is fundamental when making informed clinical decisions and treatment strategies (Norris et al., 2020). More so, regarding ANS function, there are known chromosomal sex differences in HRV and BP dynamics (Koenig & Thayer, 2016). Specifically, despite a higher resting HR, females display higher PNS sensitivity compared to males, and parallelly, males seem to be more sensitive to SNS activity despite a lower resting HR (Koenig & Thayer, 2016). Particular with exercise, some investigation into sex differences in ANS recovery has been completed (Humm et al., 2021; Kappus et al., 2015; Mendonca et al., 2010; Queiroz et al., 2013), although few are specific to resistance exercise (Humm et al., 2021) with a paucity of studies including sustained, cyclical resistance exercise (>1‐min).

With the aforementioned in mind, the primary objective of this investigation was to investigate if load intensity impacted ANS function (HRV, BRS) during and immediately following (< 15‐min) bodyweight SSM (bSSM) and resisted SSM (rSSM) with 10% of one's bodyweight held in each arm (20% bodyweight total). The secondary objective was to determine the extent chromosomal sex impacted the above relationship. Based on the described literature, it was hypothesized that (1) rSSM would elicit greater cardiovascular stress compared to bSSM that would translate to differences in autonomic function (HRV and BRS) during and immediately following the maneuvers, and (2) there would be differences in autonomic function during and following both SSM between chromosomal sexes.

## 2 MATERIALS AND METHODS

### 2.1 Ethics

The Conjoint Health Research Ethics Board at the University of Calgary (REB20‐2112) approved this study under a larger project examining how resistance SSM maneuvers impacted both autoregulatory as well as autonomic function in humans. Written informed consent was provided by all participants and all protocols were thoroughly explained and detailed prior to collection. Data were collected in July of 2021 and all procedures were followed according to REB20‐2112 guidelines.

### 2.2 Participants

A convenience sample of 25 healthy adults (13 females and 12 males) with moderate‐to‐high levels of fitness were recruited to participate in this study. Females were an average age of 21.8 ± 2.2 years, with a mean weight of 66.8 ± 12.8 kg, and a mean height of 164.8 ± 6.2 cm. Males were an average age of 24.6 ± 2.8 years, had a mean weight of 84.8 ± 17.9 kg, and a mean height of 177 ± 8.3 cm. Full demographic information of participants is displayed in Table 1. Participants had no history of cerebrovascular, cardiorespiratory, musculoskeletal, and/or neurological conditions. Instruction was given to abstain from alcohol and caffeine for a minimum of 8‐hours prior to data collection (Ainslie et al., 2007, 2008; Smirl et al., 2014, 2015). Participants were also instructed to refrain from participating in exercise for at least 6‐hours prior to data collection (Burma et al., 2020). The exercise guideline was determined using a conservative approach, as it has been shown that ANS recovery following exercise occurs ~1‐h following exercise (Burma et al., 2020; Seiler et al., 2007). Additionally, participants were instructed to refrain from food consumption for 2‐hours prior to data collection to mitigate any potential ANS influences associated with carbohydrate absorption (Baak, 2008; Burma et al., 2020).

**TABLE 1:** Participant demographics and environmental testing conditions in 25 individuals (13 females/12 males)

	Sex	Mean ±SD	Sex Cohen's *d*
**Demographic**			
Age (years)	Female	21.8 ± 2.2	*d* = 1.08
	Male	24.6 ± 2.8	
	Total	23.2 ± 2.8	
BMI (kg/m^2^)	Female	24.5 ± 3.6	*d* = 0.58
	Male	26.9 ± 4.6	
	Total	25.7 ± 4.2	
Weight (kg)	Female	66.8 ± 12.8	*d* = 1.16
	Male	84.8 ± 17.9	
	Total	75.4 ± 17.7	
Height (cm)	Female	164.8 ± 6.2	*d* = 1.67
	Male	177.0 ± 8.3	
	Total	170.6 ± 9.5	
**Environmental factors**			
Forehead temperature (°C)	Female	36.5 ± 0.3	*d* = 0.04
	Male	36.6 ± 0.3	
	Total	36.6 ± 0.3	
Calgary barometric pressure (mmHg)	Female	667.7 ± 1.8	*d* = 0.73
	Male	669.4 ± 2.7	
	Total	668.5 ± 2.4	
Room humidity (%)	Female	48.6 ± 11.3	*d* = 0.01
	Male	48.8 ± 13.7	
	Total	48.7± 12.2	
Room temperature (°C)	Female	21.1 ± 0.2	*d* = 0.19
	Male	21.0 ± 0.4	
	Total	21.0 ± 0.3	
Resistance weight relative to bodyweight (20% bodyweight;10% per hand) during SSM (%)	Female	20.1 ± 2.3	*d* = 0.10
	Male	19.9 ± 1.0	
	Total	20.0 ± 1.8	

Note

Data are presented as mean ± standard deviation. Cohen's *d* were computed with thresholds of <0.2, 0.2–0.5, 0.5–0.8 and >0.8 to quantify negligible, small, moderate, and large effect, respectively. Kilograms per meters squared (kg/m^2^), kilograms (kg), centimeters (cm), degrees Celsius (°C), milimeters of mercury (mmHg), percent (%).

### 2.3 Instrumentation

Heart activity measures (R‐R intervals) were obtained using a three‐lead electrocardiogram (ECG), via lead II methodology (Meek & Morris, 2002) (FE 231: ADInstruments, Colorado Springs CO, USA). Leads were attached such that one was placed on the top right and left of the participants’ chest (inferior to the collar bone), and one was placed on the right side of the lower torso (in line with the umbilicus) (Meek & Morris, 2002). Finger photoplethysmography placed over the third intermediate phalanx was used to obtain beat‐to‐beat BP data (Finapres NOVA: Finapres Medical Systems, Amsterdam, The Netherlands) (Omboni et al., 1993; Sammons et al., 2007). This also contained a height calibration unit, which has shown to be reliable for correcting BP values to the level of the heart (Omboni et al., 1993; Sammons et al., 2007). The respiration rate was monitored through a mouthpiece and an inline gas analyzer (ML 206: ADInstruments, Colorado Springs CO, USA). Participants wore a nose clip to prevent nasal ventilation. Prior to the rSSM, Bowflex 552 dumbbells (Nautilus I10nc, Vancouver WA, USA) were calibrated to 10% bodyweight (nearest 2.5 lbs) and supported with each arm (20% bodyweight total). Twenty percent of individuals’ bodyweight as overall resistance was used in order to ensure 5‐min of SSM could be completed (importance outlined below). To allow for robust BP and ECG waveform data, weightlifting hooks were strapped to participants’ wrists (Iron Bull Strength, Sherbrooke, QC, CAN). This enabled participants’ hands, arms, and torso to stay relaxed while holding the weights, ensuring uninterrupted BP and ECG recordings.

### 2.4 Experimental protocol

Participants underwent testing during a single laboratory visit at the University of Calgary Cerebrovascular Concussion Lab. Environmental conditions of the laboratory were measured, including barometric pressure of 668.6 ± 3.1 mmHg, humidity at 48.7 ± 12.2%, and temperature of 21 ± 0.3 °C (Table 1). The laboratory was located at 1111 m above sea level. All testing occurred over the course of an individual's normal workday, where HRV and BRS research has shown to be nominally impacted by diurnal variation when collected in an upright standing posture (Burma et al., 2020).

Upon entering the laboratory, height, weight, and demographic information were recorded and equipment was fitted for each participant. Participants then performed two bouts of bSSM, occurring at 0.05 Hz (10‐s squat–10‐s stand; 20‐s total per cycle) and 0.10 Hz (5‐s squat–5‐s stand; 10‐s total per cycle) (Smirl et al., 2015). The order of the SSM frequencies performed were randomized across participants and chosen as frequencies of 0.05 and 0.10 Hz, parallel with naturally occurring BP maintenance processes–metabolic, endothelial, neurogenic, and/or myogenic factors (0.05 Hz) and sympathetic influences (0.10 Hz) (Hamner & Tan, 2014; Tan et al., 2013). As stated, this was a subsection of a larger investigation examining how cerebrovascular and autonomic measures are impacted by resistance exercise, with the cerebrovascular data published elsewhere (in submission). Participants performed these maneuvers for a duration of 5‐minutes, as this is required to derive valid and reliable time‐ and frequency‐domain short‐term HRV metrics (Bourdillon et al., 2017; Burma, Graver, et al., 2021; Schroeder et al., 2004). Immediately after the two bouts of bSSM, participants moved to a quiet seated position for 5‐min, followed by 5‐min of quiet standing. Participants were instructed to remain as still as possible and to breathe normally during each of the sitting and standing tasks. A minimum 1‐min period of BP normalization was allotted before the beginning of the 5‐minute standing task (after transitioning from sitting to standing), albeit this was extended as required to ensure steady‐state measures of BP and HR were present and minimize the potential influence of the postural change on the ANS measures of interest (Harms et al., 2021).

Following a rest period of ~30–60 min, the same protocol was repeated with additional resistance equalling ~20% of participants’ bodyweight (~10% held in each hand) during the SSM. This resistance amount was strategically chosen based on methodological considerations and pilot testing by the researchers. For example, performing near‐maximal weight resistance exercise elicits substantial MAP oscillations (>200 mmHg). However, at this intensity individuals are typically only able to complete 10 or fewer repetitions (depending on the relative weight). With such a stressor, one would not be able to derive meaningful/robust HRV estimates. Therefore, the research group piloted various resistances of one's total body mass (e.g., 10%, 20%, 30%, 40%, and 50%) to ensure the selected resistance sufficiently stressed the cardiovascular system, while being a stimulus that participants could hold for ~5‐min (Burma, Graver, et al., 2021; Burma, Miutz, et al., 2021). Conclusively, 20% was utilized as this was the highest amount of resistance applied that elicited meaningful data.

The order of SSM completion followed a block design in which the bSSM block occurred before the rSSM block. This design, in conjunction with ample recovery time (~30–60 min) between SSM tasks, was utilized instead of randomization as it better minimized any effect the first sets of SSM would have on the following sets. Specifically, bSSM were performed first as the required recovery from such would likely be less than that of rSSM, reducing the chance of any autonomic alterations due to the first set of SSM impacting that of the following set. The time interval between bSSM and rSSM (~30–60 min) was chosen in accordance with ANS recovery occurring, in under 60‐min following mild‐to‐moderate exercise (Burma et al., 2020; Seiler et al., 2007). Lastly, as a safeguard, physiological and autonomic variables, specifically MAP and HR, were ensured to be comparable to normal baseline values prior to the start of the rSSM block.

### 2.5 Outcome measures

Primary outcome measures of autonomic function were HRV and BRS. Heart rate variability describes the fluctuations between consecutive heartbeats and is commonly categorized in time‐ and frequency‐domains (Shaffer & Ginsberg, 2017). The former measures HRV through differences in milliseconds (ms) regarding the between beat R‐R interval variation (Shaffer & Ginsberg, 2017). Time domain metrics analyzed in this study are HR, a standard deviation of successive R‐R intervals (SDNN), root mean square of the successive R‐R intervals (RMSSD), and percent of R‐R intervals that differed by >50 ms (pNN50). Higher values for SDNN, RMSSD, and pNN50 suggest parasympathetic dominance, whereas lower values indicate sympathetic dominance (Shaffer & Ginsberg, 2017). Frequency‐domain measures use power spectral density to breakdown HRV into its frequency components and their respective intensities (e.g., low‐frequency [LF]: 0.04–0.15 Hz and high‐frequency [HF]: 0.15–0.40 Hz) (Shaffer & Ginsberg, 2017). The HF band is thought to be predominately controlled by the PNS (Malik et al., 1996), whereas the LF band is thought to primarily represent SNS activity (Pagani et al., 1986) with a small degree of input from the PNS (Randall et al., 1991). Generally, high HRV at rest is associated with PNS dominance and healthy ANS function, while low HRV at rest is associated with SNS dominance and has been linked with clinical conditions such as coronary heart disease, anxiety, and sudden cardiac death (Chalmers et al., 2014; Dekker et al., 2000; Malik et al., 1996; Sessa et al., 2018; Xhyheri et al., 2012). Baroreceptor sensitivity is the intrinsic ability of the heart to respond according to changes in BP (HR change per mmHg change in BP; ms/mmHg) (Swenne, 2013). It acts in a negative feedback loop and is commonly characterized by low‐frequency gain, which is calculated by dividing RR interval power spectral density (PSD) by systolic BP PSD (Swenne, 2013). Typically, higher BRS low‐frequency gain coincides with better health, as a strong feedback loop exists between the carotid/aortic baroreceptors and the cardiac fibers (Parati et al., 2001) to aid in the maintenance of homeostasis. Both HRV and BRS are established measures of autonomic nervous system function and cardiovascular health (Acharya et al., 2006; La Rovere et al., 2008).

### 2.6 Data analysis

HRV metrics were derived from the ECG R‐R intervals collected during the 5‐minute recordings of each task. Extracted data were visually inspected and manually corrected such that misaligned and artifact R‐spikes were filtered and normalized. Artifacts occurred in less than <0.5% of QRS complexes. Data were then processed and analyzed via commercially available software (Ensemble‐R V1.0.42, R&D Canvas, Wellington, NZ). Data were also subjected to fast Fourier transformation to obtain BRS PSD, BRS gain, and frequency‐domain HRV metrics: LF (0.04–0.15 Hz) and HF (0.15–0.40 Hz). In summary, time‐domain HRV output variables were HR, SDNN, RMSSD, and pNN50. Frequency‐domain HRV output variables included relative LF, relative HF, and LF/HF. Absolute frequency‐domain metrics were not considered due to their poor reliability and validity during short‐term HRV recordings (Burma, Graver, et al., 2021). Baroreceptor sensitivity outcome measures were derived at 0.10 Hz during the sitting and standing protocols, and at the point estimate of interest during the SSM (0.05 and 0.10 Hz), which included RR interval PSD, systolic BP PSD, and the associated gain metric 0.05 Hz (0.05 Hz SSM) and 0.10 Hz (0.10 Hz SSM, quiet‐sitting, and quiet‐standing).

### 2.7 Sample size calculation

The sample size required to answer the *a priori* research questions for the present investigation was determined from a previous study (Burma et al., 2020) through G*power software (v3.1.9), which examined the extent to which both high‐intensity intervals and moderate‐intensity continuous exercise impacted time‐ and frequency‐domain HRV metrics, as well as BRS LF gain. The present study employed a low‐level resistance exercise stimulus, opposed to moderate‐ and high‐intensity aerobic exercise. However, the current investigation examined HRV and BRS metrics within 10‐min following the exercise stimulus, as opposed to Burma et al., (Burma et al., 2020), which examined the same metrics ~10–15‐min post‐exercise. Previous research has demonstrated, substantial recovery occurs within the first 10‐min following low‐, moderate‐, and high‐intensity aerobic exercise, albeit in a dose‐response relationship (Michael et al., 2017). As described, SSM produces substantial MAP oscillations and can be completed for longer durations compared to typical resistance exercises. Hence, the ANS oscillations associated with rSSM data may be more symmetrical to HRV/BRS changes with interval aerobic exercise compared to continuous exercise. Data from Burma et al. (2020) were collected ~10–15‐min post‐exercise for both high‐and moderate‐intensity conditions; however, it can be speculated that differences in exercise intensity may elicit variations in the physiological response to activity compared to SSM. Therefore, to determine the sample size calculation required for the present investigation, values from (Burma et al., 2020) were averaged between the moderate‐intensity continuous exercise and the high‐intensity interval exercise, rather than using either independently. The justification for this includes the latter exercise inducing autonomic oscillations due to the nature of interval exercise, whereas the inclusion of the former controls for the lower‐level intensity. More so, as HRV/BRS values were collected ~10–15‐min post‐exercise in Burma et al. (2020), it is thought this would resemble that of the bSSM and rSSM immediately following the completion of these maneuvers (0‐min). To compute the effect size required for the current investigation, the average exercise values from both conditions were combined and compared to baseline pre‐exercise values in Burma et al. (2020). It is important to note that the HRV/BRS measures were significantly different following the high‐intensity intervals but not the moderate‐intensity exercise. This means the effect sizes were derived based upon significant and null results, enabling for a precise estimate of the required sample size. These data points were then combined for all variables, which produced an effect size (eta squared) of 0.285 and a correlation of (*r* = 0.60). Using these values with an alpha of 0.05, it was determined that a sample of 18 participants was required to achieve a power of 0.80. Finally, an investigation by Mendonca et al. (2010) examined chromosomal sex differences in HRV recovery following a 30‐s Wingate in 13 males and 12 females. Using the differences in sexes of the relative frequency‐domain metrics following the exercise, an eta squared effect size of 0.255 was produced. Using the same alpha and power thresholds, it was determined that 20 participants total were required to determine differences in sexes regarding ANS recovery.

### 2.8 Statistical analysis

RStudio (v1.2.5042) was used to perform all statistical analyses. Differences in absolute cardiovascular and respiratory variables between all completed SSM were assessed using a 2 × 4 Analysis of Variance (ANOVA). This enabled for an understanding if the type of SSM and/or sex impacted the physiological values and was not stratified by frequency, to understand if a given frequency produced a different physiological response during the bSSM and rSSM. Prior to all comparisons, Levene's test was used to assess the homogeneity of variance between groups. If this was violated, and/or the data within a group contained the presence of an extreme outlier, the data for a given comparison were log‐transformed to minimize the likelihood of making a Type I error. For all HRV and BRS outcomes measures, task (bSSM versus rSSM) and chromosomal sex (female vs. male) comparisons were completed via an omnibus repeated measures ANOVA (2 × 2). This approach has been proven valid and reliable for between group comparisons with the sample size is ≥15 participants, despite having equal or unequal variances and sample sizes between groups (Blanca et al., 2017). Moreover, if significant differences across task and/or sex were calculated, Tukey's Honestly Significant Difference post‐hoc pairwise comparisons were completed to delineate specific group differences. Effect sizes from the ANOVA models were calculated using generalized eta squared (η^2^ *_G_*) coefficient. Thresholds of <0.02, 0.02–0.13, 0.13–0.26, and >0.26 were used to quantify negligible, small, moderate, and large effect size, respectively (Bakeman, 2005). For the post hoc comparisons, Cohen's *d* values were also computed with thresholds of <0.2, 0.2–0.5, 0.5–0.8, and >0.8 were used to quantify negligible, small, moderate, and large, respectively (Lakens, 2013). Effect sizes were computed to account for recent concerns within physiological/biomedical literature regarding the sole reporting of *p*‐*values*, especially when making inferences based on a binary threshold (Amrhein et al., 2019; Panagiotakos, 2008). Therefore, within the present investigation, all inferences were made based on the combination of *p*‐*values* and effect sizes. Within‐subject coefficient of variation (CoV) metrics were computed to demonstrate the physiological variation between tasks. These were calculated as a percentage of the standard deviation over the mean for each measure (Atkinson & Nevill, 1998). To determine the 95% confidence intervals for the CoV metrics, bootstrap analyses were performed where 10,000 resamples were conducted on the calculated CoV values for each subject. Alpha was determined *a priori* at 0.05. All data were presented as mean ± standard deviation or mean ±95% confidence interval, where appropriate.

## 3 RESULTS

### 3.1 General physiaological variables main effects during and following squat‐stand maneuvers

Absolute cardiovascular and respiratory variables during the SSM tasks and during immediate recovery from the SSM are displayed in Tables 2 and 3, respectively. During SSM, a task main effect was found for diastolic BP (*F*_(3,92)_ = 12.3; *p* < 0.001; $\eta_{G}^{2}$ = 0.29 [large]) and MAP (*F*_(3,92)_ = 11.1; *p* < 0.001; $\eta_{G}^{2}$ = 0.29 [large]). Further, a sex main effect was found for systolic BP (*F*_(1,92)_ = 7.10; *p* = 0.009; $\eta_{G}^{2}$ = 0.07 [small]). No other task (all *F*_(3, 92)_ < 1.92, all *p* > 0.132; all $\eta_{G}^{2}$ < 0.06 [negligible/small]), sex (all *F*_(3, 92)_ < 3.88, all *p* > 0.052; all $\eta_{G}^{2}$ < 0.04 [negligible/small]) or task by sex (all *F*_(3, 92)_ < 2.55, all *p* > 0.061; all $\eta_{G}^{2}$ < 0.08 [negligible/small]) main effects were present. During the quiet sitting and quiet standing tasks (post‐SSM), no general physiological main effects were found (all *F*_(3, 92)_ < 2.55, all *p* > 0.061; all $\eta_{G}^{2}$ < 0.08 [negligible/small]) (Table 3). Notably, the largest CoV between all four SSM were within diastolic BP and MAP metrics (Table 2).

**TABLE 2:** Cardiovascular and respiratory variables during SSM at 0.05 and 0.10 Hz in 25 individuals (13 females/12 males)

	Post‐SSM	Post‐rSSM	CoV (95% CI)
**0.05 Hz**			
P_ET_CO2 (mmHg)	39.1 ± 3.4	39.2 ± 3.1	4.1% (1.9–3.2%)
Female	38.9 ± 3.3	38.6 ± 2.9	4.0% (2.4–5.6%)
Male	39.3 ± 3.7	40.0 ± 3.3	4.2% (2.3–6.0%)
Respiratory rate (BPM)	16.6 ± 4.3	17.3 ± 4.5	9.6% (7.3–12.0%)
Female	16.2 ± 4.7	16.9 ± 5.0	10.5% (7.2–13.8%)
Male	17.0 ± 3.9	17.7 ± 4.1	8.7% (5.5–11.9%)
Systolic arterial pressure (mmHg)	129.9 ± 16.9	139.4 ± 20.0	8.5% (6.3–10.7%)
Female	125.4 ± 21.0	136.2 ± 10.0	10.7% (7.4–14.1%)
Male	134.7 ± 9.70	142.9 ± 14.4	6.1% (4.1–8.2%)
Diastolic arterial pressure (mmHg)	61.7 ± 10.3	76.3 ± 11.1	17.0% (13.3–20.7%)
Female	63.4 ± 13.2	79.5 ± 13.4	19.9% (14.4–25.5%)
Male	59.8 ± 5.60	72.9 ± 7.1	13.9% (9.9–18.0%)
Mean arterial pressure (mmHg)	81.9 ± 11.1	96.6 ± 12.1	13.1% (10.1–16.2%)
Female	82.9 ± 14.7	98.8 ± 15.3	15.2% (10.4–20.0%)
Male	80.8 ± 5.50	94.3 ± 7.3	10.9% (7.7–14.2%)
**0.10 Hz**			
P_ET_CO2 (mmHg)	39.9 ± 3.7	39.7 ± 3.8	4.9% (3.3–6.4%)
Female	39.0 ± 3.2	39.5 ± 4.0	5.8% (3.6–8.0%)
Male	40.9 ± 4.0	40.0 ± 3.8	3.9% (1.9–5.9%)
Respiratory rate (BPM)	16.3 ± 4.5	17.5 ± 5.1	10.8% (7.9–13.7%)
Female	16.2 ± 5.4	17.2 ± 5.9	9.6% (5.0–14.1%)
Male	16.4 ± 4.5	17.8 ± 4.2	12.2% (8.6–15.7%)
Systolic arterial pressure (mmHg)	132.0 ± 17.5	139.7 ± 19.6	7.7% (5.1–10.4%)
Female	127.8 ± 20.8	132.9 ± 17.6	9.4% (5.2–13.4%)
Female	136.6 ± 12.4	147.0 ± 19.7	6.0% (3.1–9.0%)
Diastolic arterial pressure (mmHg)	60.9 ± 11.6	72.9 ± 11.6	16.3% (11.9–20.1%)
Female	62.7 ± 13.4	74.7 ± 13.5	18.9% (12.7–25.1%)
Male	59.0 ± 9.3	71.0 ± 9.4	13.5% (7.9–19.2%)
Mean arterial pressure (mmHg)	81.9 ± 11.5	94.4 ± 12.1	12.6% (9.4–15.8%)
Female	83.2 ± 14.3	94.5 ± 13.8	14.0% (8/9–19.1%)
Male	80.5 ± 8.0	94.3 ± 10.7	11.0% (7.4–14.7%)

Note

Values are mean ± standard deviation.

Abbreviations: bpm, beats per minute; BPM, breaths per minute; mmHg, millimeters of mercury; P_ET_CO_2_, End tidal values of carbon dioxide; RR, respiratory rate.

The coefficient of variation (CoV) values were calculated using the mean values from each subject, where a bootstrap approach with 10,000 resamples.

**TABLE 3:** Cardiovascular and respiratory variables during the sitting and standing recovery periods in 25 individuals (13 females/12 males)

	Post‐SSM	Post‐rSSM	CoV (95% CI)
**Sitting**			
P_ET_CO2 (mmHg)	38.5 ± 3.9	38.7 ± 4.2	4.2% (3.0–5.3%)
Female	38.0 ± 3.7	38.7 ± 3.7	3.3% (1.9–4.7%)
Male	39.0 ± 4.1	38.7 ± 4.7	5.1% (3.4–6.8%)
Respiratory rate (BPM)	13.3 ± 4.0	13.5 ± 4.0	5.3% (3.5–7.0%)
Female	13.1 ± 4.4	13.4 ± 4.4	4.9% (2.5–7.2%)
Male	13.4 ± 3.9	13.7 ± 3.7	5.7% (3.2–8.3%)
Systolic arterial pressure (mmHg)	115.3 ± 18.2	124.7 ± 15.9	7.5% (5.2–9.8%)
Female	114.6 ± 22.6	123.9 ± 19.1	7.8% (4.3–11.3%)
Male	116.0 ± 12.7	125.5 ± 12.2	6.7% (2.9–10.5%)
Diastolic arterial pressure (mmHg)	63.2 ± 10.6	66.3 ± 7.2	9.5% (6.2–12.7%)
Female	64.6 ± 13.3	66.0 ± 8.0	6.1% (4.7–7.5%)
Male	61.7 ± 6.90	66.6 ± 6.5	5.2% (4.1–6.2%)
Mean arterial pressure (mmHg)	79.3 ± 12.8	84.2 ± 8.4	7.9% (5.5–10.4%)
Female	79.4 ± 16.3	84.8 ± 10.1	12.0% (7.2–16.8%)
Male	79.3 ± 8.10	83.5 ± 6.4	5.0% (2.7–7.2%)
**Standing**			
P_ET_CO2 (mmHg)	37.0 ± 3.3	36.2 ± 3.4	3.6% (2.6–4.7%)
Female	36.2 ± 3.4	35.7 ± 3.7	3.4% (2.2–4.7%)
Male	37.8 ± 3.1	36.6 ± 3.2	3.8% (2.1–5.5%)
Respiratory rate (BPM)	12.6 ± 4.2	12.8 ± 4.2	4.8% (3.3–6.3%)
Female	11.6 ± 4.4	12.2 ± 4.7	5.3% (3.2–7.5%)
Male	13.7 ± 3.9	13.3 ± 3.9	4.2% (2.3–6.1%)
Systolic arterial pressure (mmHg)	116.3 ± 19.1	123.3 ± 19.9	8.1% (6.0–10.1%)
Female	115.2 ± 23.9	124.1 ± 20.5	7.9% (4.8–11.1%)
Male	117.3 ± 13.0	122.5 ± 20.0	8.2% (5.6–10.8%)
Diastolic arterial pressure (mmHg)	67.7 ± 11.7	70.8 ± 9.1	10.6% (7.4–13.9%)
Female	68.8 ± 14.8	70.1 ± 10.0	13.0% (8.1–17.8%)
Male	66.4 ± 7.70	71.5 ± 8.4	8.1% (4.4–11.8%)
Mean arterial pressure (mmHg)	83.1 ± 13.3	87.6 ± 11.1	9.0% (6.6–11.4%)
Female	83.6 ± 16.8	87.4 ± 11.0	10.6% (7.2–14.0%)
Male	82.7 ± 8.90	87.8 ± 11.7	7.2% (4.2–10.3%)

Note

Values are mean ± standard deviation.

Abbreviations: bpm, beats per minute; BPM, breaths per minute; mmHg, millimeters of mercury; P_ET_CO_2_, End tidal values of carbon dioxide; RR, respiratory rate.

The coefficient of variation (CoV) values were calculated using the mean values from each subject, where a bootstrap approach with 10,000 resamples.

### 3.2 General physiological variables post hoc comparisons during and following squat‐stand maneuvers

Post hoc analysis revealed diastolic BP (all *p* < 0.003, Cohen's *d* > 1.03 [large]) and MAP (all *p* < 0.003, Cohen's *d* > 1.03 [large]) were higher during rSSM compared to bSSM at both 0.05 and 0.10 Hz frequencies (Tables 2 and 3). Regarding task differences, males had higher systolic BP (*p* = 0.009; Cohen's *d* = 0.52 [moderate]) (Tables 2 and 3).

### 3.3 HRV and BRS main effects during squat‐stand maneuvers

**FIGURE 1:**
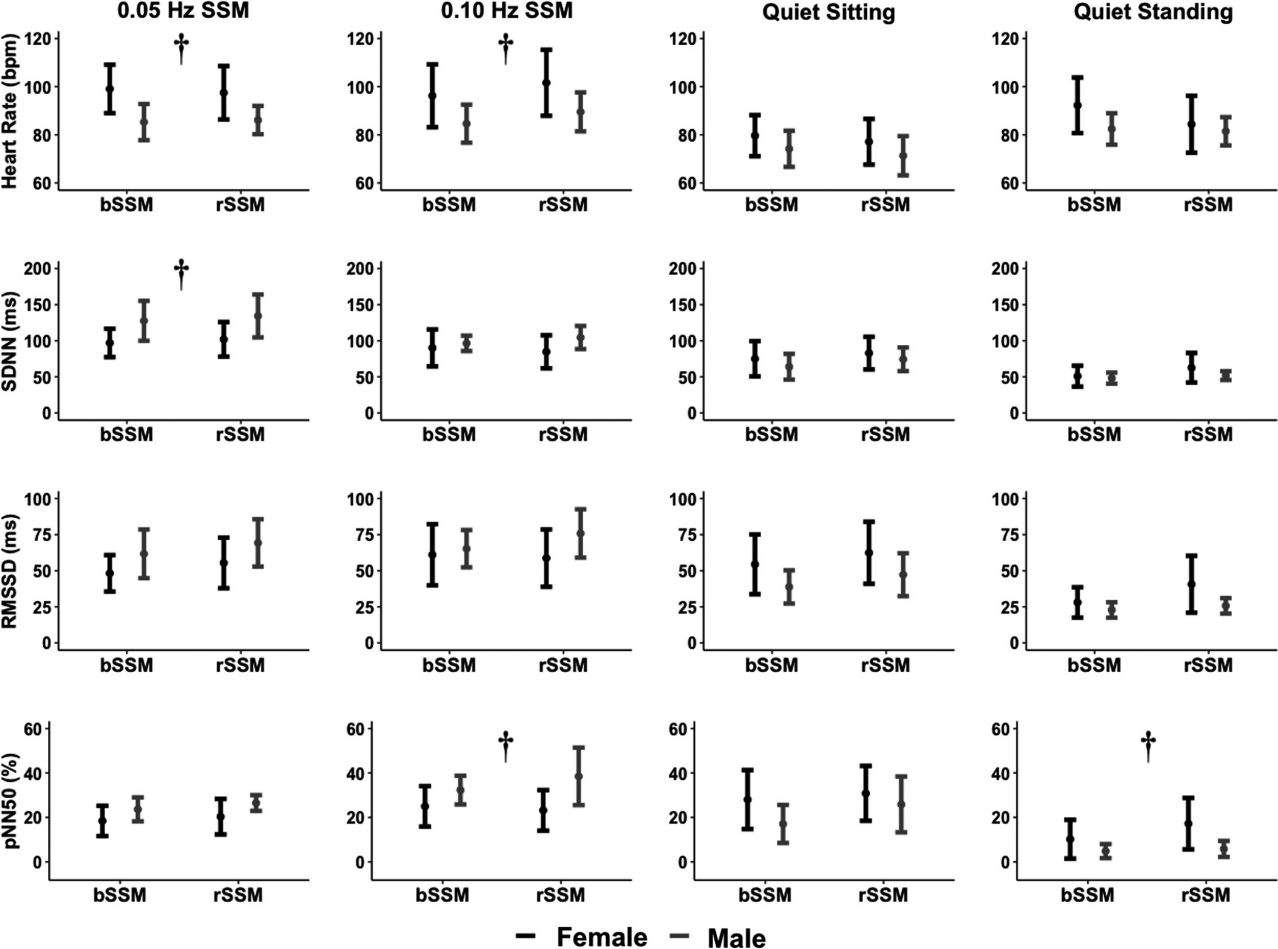
Time‐domain HRV metrics including heart rate (BPM), standard deviation of successive R‐R intervals (SDNN; ms), root mean square of the successive R‐R intervals (RMSSD; ms), and percent of R‐R intervals that differed by >50 ms (pNN50; %). Metrics are displayed for both sexes across task type (bodyweight squat‐stand maneuver [bSSM] and resistance squat‐stand maneuver [rSSM]) for both SSM task frequencies (0.05 and 0.10 Hz) as well as quiet sitting recovery and quiet standing recovery tasks. Task (bSSM vs. rSSM) and chromosomal sex (female vs. male) comparisons were completed via an omnibus two‐factorial repeated measures ANOVA. Post‐hoc comparisons were performed using Tukey's honestly significant difference pairwise comparisons with Cohen's *d* effect sizes. Dagger (†) denotes significant sex main effect. No task main effects were found. Milliseconds (ms), beats per minute (bpm), and percent (%)

**FIGURE 2:**
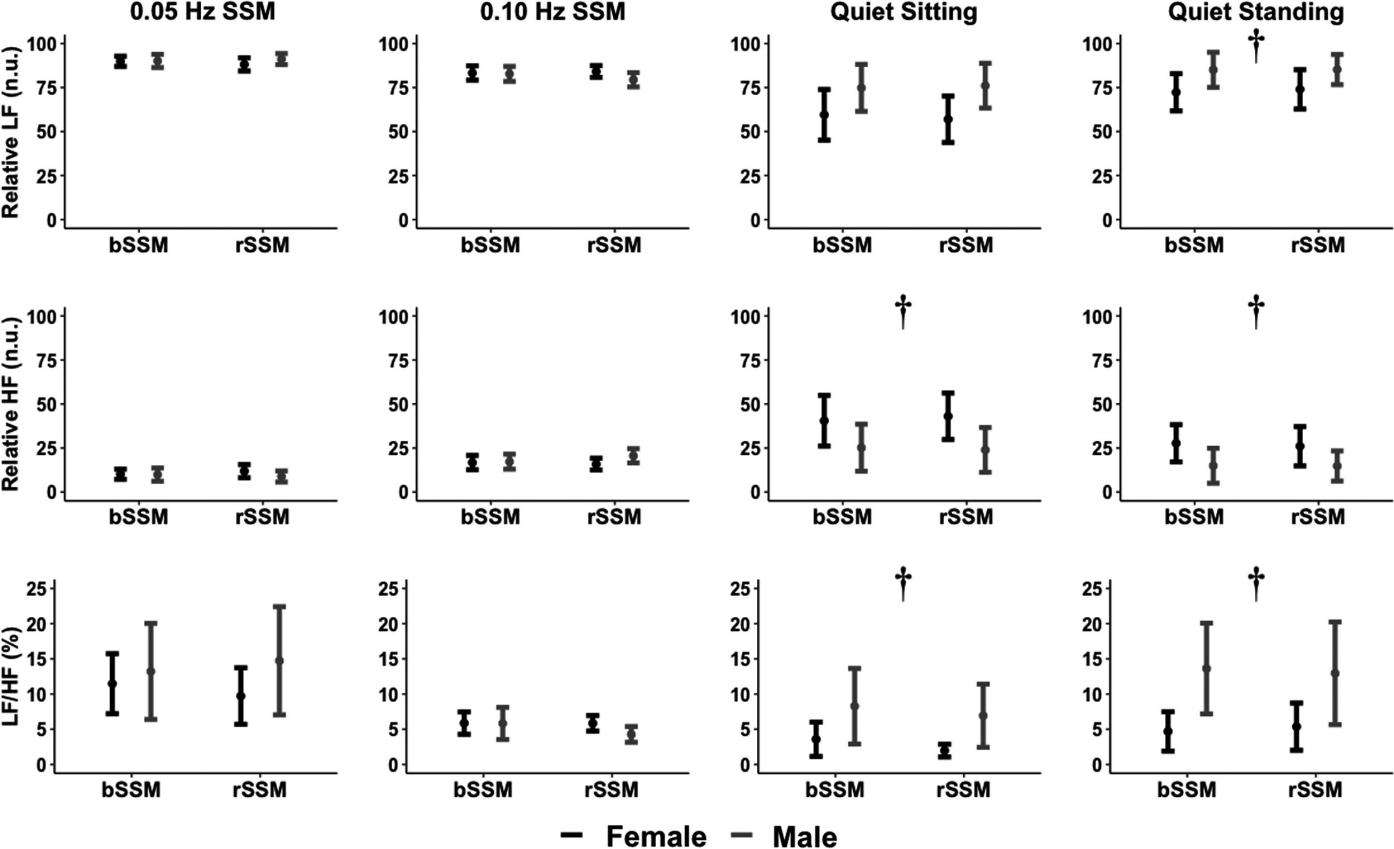
Frequency‐domain HRV metrics including relative low‐frequency (LF; n.u.), relative high‐frequency (HF; n.u.), and relative low‐frequency to high‐frequency ratio (LF/HF; %). Metrics are displayed for both sexes across task type (bodyweight squat‐stand maneuver [bSSM] and resistance squat‐stand maneuver [rSSM]) for both SSM task frequencies (0.05 and 0.10 Hz) as well as quiet sitting recovery and quiet standing recovery tasks. Task (bSSM vs. rSSM) and chromosomal sex (female vs. male) comparisons were completed via an omnibus two‐factorial repeated measures ANOVA. Post‐hoc comparisons were performed using Tukey's honestly significant difference pairwise comparisons with Cohen's *d* effect sizes. Dagger (†) denotes significant sex main effect. No task main effects were found. Normalized units (n.u.) and percent (%)

**FIGURE 3:**
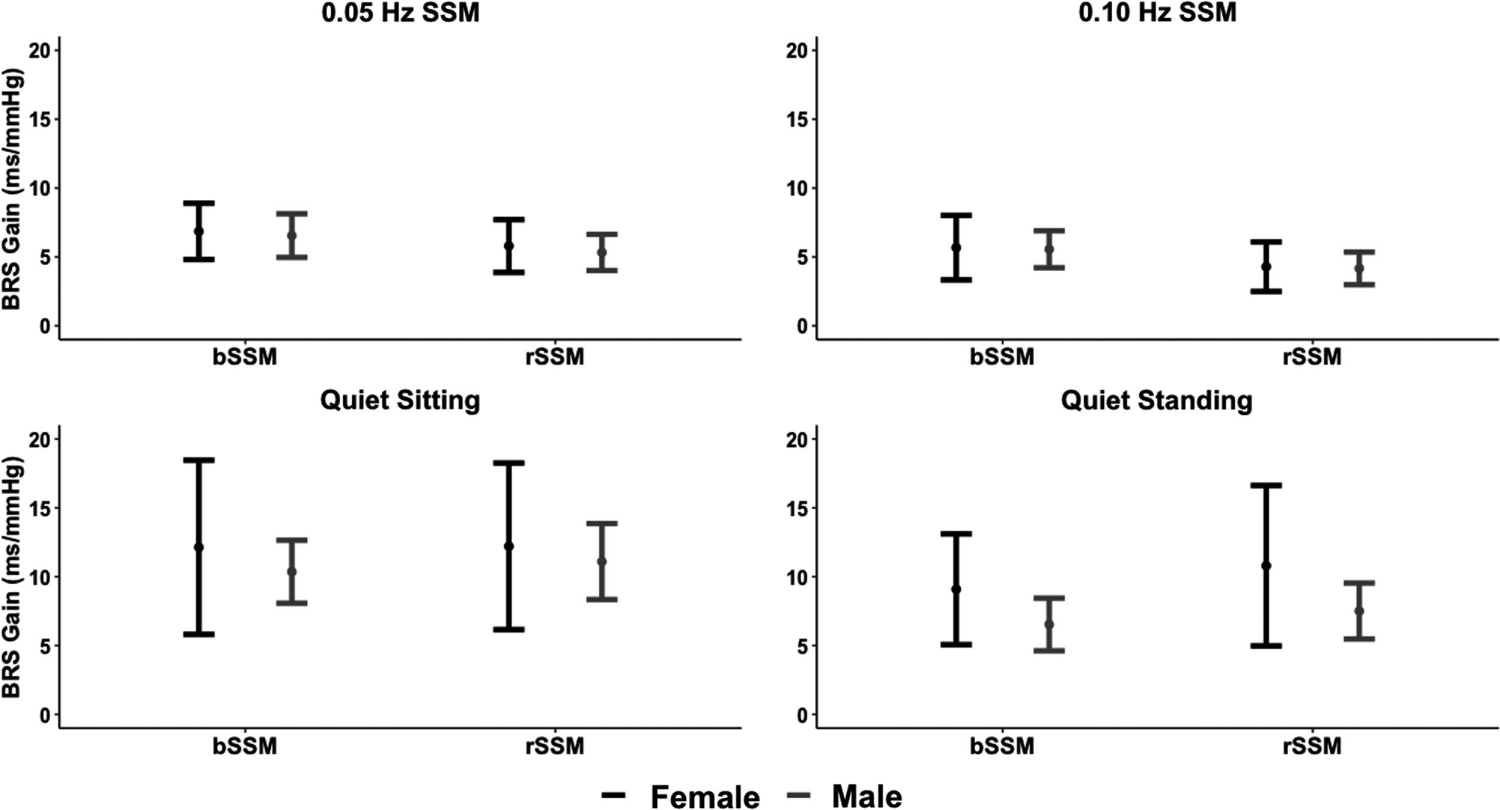
Baroreceptor sensitivity metrics including low‐frequency baroreceptor sensitivity (BRS) gain (ms/mmHg). Baroreceptor sensitivity gain is calculated by change in heart rate (ms) divided by change in blood pressure (mmHg) Metrics are displayed for both sexes across task type (bodyweight squat‐stand maneuver [bSSM] and resistance squat‐stand maneuver [rSSM]) for both SSM task frequencies (0.05 and 0.10 Hz) as well as quiet sitting recovery and quiet standing recovery tasks. Task (bSSM vs. rSSM) and chromosomal sex (female vs. male) comparisons were completed via an omnibus two‐factorial repeated measures ANOVA. Post‐hoc comparisons were performed using Tukey's honestly significant difference pairwise comparisons with Cohen's *d* effect sizes. No sex or task main effects were found for BRS metrics. Milliseconds (ms) and milometers of mercury (mmHg)

A sex main effect was found for HR at 0.05 Hz (*F*_(1, 44)_ = 8.81; *p* = 0.005; $\eta_{G}^{2}$ = 0.17 [moderate]) and 0.10 Hz (*F*_(1, 44)_ = 5.04; *p* = 0.030; $\eta_{G}^{2}$ = 0.10 [small]) (Figure 1). Further, sex main effects were present during 0.05 Hz SSM for SDNN (*F*_(1, 44)_ = 7.64; *p* = 0.008; $\eta_{G}^{2}$ = 0.15 [moderate]) (Figure 1) and RR interval power spectral density (*F*_(1, 44)_ = 7.04; *p* = 0.011; $\eta_{G}^{2}$ = 0.14 [moderate]) (Table 4). Sex main effects were also found for pNN50 (*F*_(1,44)_ = 6.65; *p* = 0.013; $\eta_{G}^{2}$  = 0.13 [moderate]) (Figure 1) and systolic BP power spectral density (*F*_(1,44)_ =4.50; *p* = 0.039; $\eta_{G}^{2}$  = 0.09 [small]) (Table 4) during the 0.10 Hz SSM. No sex main effects were found for any other HRV or BRS metric (all *F*_(1, 44)_ < 3.51; all *p* > 0.067; all $\eta_{G}^{2}$  < 0.07 [negligible/small]) (Figures 2 and 3). Additionally, no main effects for task (all *F*_(1, 44)_ < 1.04; all *p* > 0.313; all $\eta_{G}^{2}$  < 0.02 [negligible/small]) or task by sex (all *F*_(1, 44)_ < 0.14; all *p* > 0.247; all $\eta_{G}^{2}$  < 0.03 [negligible/small]) were found for any variable.

**TABLE 4:** R‐R interval and systolic power spectrum density across sex, SSM frequency, and SSM type in 25 individuals (13 females/12 males)

		Bodyweight SSM	Resistance SSM		Test statistic
**During 0.05 hz ssm**					
RRI PSD (ms^2^.Hz^−1^)	Total	1,168,416 ± 798,559	1,310,659 ± 1,038,727	Task effect	*F*(_1,44_) = 0.31; *p* = 0.578; $\eta_{G}^{2}$ = 0.01
	Female	871,084 ± 507,633	988,348 ± 709,207	*Sex effect	*F*(_1,44_) = 7.04; *p* = 0.011; $\eta_{G}^{2}$ = 0.14
	Male	1,519,809 ± 952,218	1,691,573 ± 1,258,320	Interaction effect	*F*(_1,44_) = 0.01; *p* = 0.915; $\eta_{G}^{2}$ = 0.01
SYS PSD (mmHg.Hz^−1^)	Total	37,363 ± 31,818	28,325 ± 23,829	Task effect	*F*(_1,44_) = 1.23; *p* = 0.273; $\eta_{G}^{2}$ = 0.03
	Female	33,253 ± 33,422	22,794 ± 21,224	Sex effect	*F*(_1,44_) = 1.66; *p* = 0.205; $\eta_{G}^{2}$ = 0.04
	Male	78,770 ± 84,128	56,705 ± 48,483	Interaction effect	*F*(_1,44_) = 0.04; *p* = 0.850; $\eta_{G}^{2}$ = 0.01
**During 0.10 hz ssm**					
RRI PSD (ms^2^.Hz^−1^)	Total	642,861 ± 453,863	664,196 ± 457,141	Task effect	*F*(_1,44_) = 0.03; *p* = 0.873; $\eta_{G}^{2}$ = 0.01
	Female	584,519 ± 555,106	590,325 ± 440,001	Sex effect	*F*(_1,44_) = 1.17; *p* = 0.285; $\eta_{G}^{2}$ = 0.03
	Male	711,810 ± 307,173	751,498 ± 482,545	Interaction effect	*F*(_1,44_) = 0.02; *p* = 0.899; $\eta_{G}^{2}$ = 0.01
SYS PSD (mmHg.Hz^−1^)	Total	56,007 ± 65,227	45,110 ± 37,590	Task effect	*F*(_1,44_) = 0.54; *p* = 0.468; $\eta_{G}^{2}$ = 0.01
	Female	36,745 ± 37,144	35,299 ± 22,845	*Sex effect	*F*(_1,44_) = 4.50; *p* = 0.039; $\eta_{G}^{2}$ = 0.09
	Male	78,770 ± 84,128	56,705 ± 48,483	Interaction effect	*F*(_1,44_) = 0.48; *p* = 0.494; $\eta_{G}^{2}$ = 0.01
**Post‐ssm quiet sitting**					
RRI PSD (ms^2^.Hz^−1^)	Total	22,038 ± 20,032	82,733 ± 158,356	Task effect	*F*(_1,46_) = 3.47; *p* *= 0.069*; $\eta_{G}^{2}$ = 0.07
	Female	25,390 ± 26,457	77,397 ± 108,636	Sex effect	*F*(_1,46_) = 0.00; *p* = 0.950; $\eta_{G}^{2}$ = 0.01
	Male	18,406 ± 9,176	88,513 ± 204,373	Interaction effect	*F*(_1,46_) = 0.08; *p* = 0.783; $\eta_{G}^{2}$ = 0.01
SYS PSD (mmHg.Hz^−1^)	Total	241 ± 299	284 ± 373	Task effect	*F*(_1,46_) = 0.21; *p* = 0.652; $\eta_{G}^{2}$ = 0.01
	Female	163 ± 245	205 ± 199	Sex effect	*F*(_1,46_) = 2.97; *p* = 0.092; $\eta_{G}^{2}$ = 0.06
	Male	325 ± 339	369 ± 495	Interaction effect	*F*(_1,46_) = 0.00; *p* = 0.991; $\eta_{G}^{2}$ = 0.01
**Post‐ssm quiet standing**					
RRI PSD (ms^2^.Hz^−1^)	Total	19,178 ± 18,672	52,002 ± 85,950	Task effect	*F*(_1,46_) = 3.61; *p* = 0.064; $\eta_{G}^{2}$ = 0.07
	Female	17,697 ± 14,923	74,704 ± 115,348	Sex effect	*F*(_1,46_) = 1.63; *p* = 0.207; $\eta_{G}^{2}$ = 0.03
	Male	20,782 ± 22,636	27,407 ± 18,275	Interaction effect	*F*(_1,46_) = 2.12; *p* = 0.152; $\eta_{G}^{2}$ = 0.04
SYS PSD (mmHg.Hz^−1^)	Total	280 ± 243	341 ± 283	Task effect	*F*(_1,46_) = 0.70; *p* = 0.409; $\eta_{G}^{2}$ = 0.02
	Female	197 ± 179	290 ± 248	Sex effect	*F*(_1,46_) = 3.65; *p* = 0.061; $\eta_{G}^{2}$ = 0.07
	Male	370 ± 277	397 ± 317	Interaction effect	*F*(_1,46_) = 0.20; *p* = 0.656; $\eta_{G}^{2}$ = 0.01

Note

Data are presented as mean ± standard deviation for males, females, and the total of both combined. The test statistics were determined through a 2 × 2 Analysis of Variance to determine main effects of type of SSM and sex. Post‐hoc comparisons were determined through Tukey's honestly significant difference. Effect sizes were determined through generalized eta squared ($\eta_{G}^{2}$), with thresholds of <0.02 (negligible), 0.02–0.13 (small), 0.13–0.26 (moderate), and >0.26 (large).

* Significant sex differences for RRI (ms^2^.Hz^−1^) during 0.05 Hz SSM and SYS PSD during 0.10 Hz SSM. Squat stand maneuvers (SSM), Hertz (Hz), Systolic (SYS), Power Spectrum Density (PSD), Milliseconds squared times hertz (ms^2^.Hz^−1^), and milometers of mercury times hertz (mmHg.Hz^−1^).

### 3.4 HRV and BRS post hoc comparisons during squat‐stand maneuvers

Post hoc analysis revealed females had a higher HR across both frequencies of SSM (Figure 1); *p* = 0.005, Cohen's *d* = 0.90 (large) and *p* = 0.030, Cohen's *d* = 0.67 (moderate) for 0.05 and 0.10 Hz, respectively. Females also displayed lower 0.10 Hz pNN50 (*p* = 0.013; Cohen's *d* = 0.75 [moderate]), 0.10 Hz systolic BP PSD (*p* = 0.040; Cohen's *d* = 0.60 [moderate]), 0.05 Hz SDNN (*p* = 0.008; Cohen's *d* = 0.81 [large]) (Figure 1) and 0.05 Hz RR interval power spectral density (*p* = 0.011; Cohen's *d* = 0.76 [moderate]) during the 0.05 Hz SSM (Table 4).

### 3.5 HRV and BRS main effects during recovery

When analyzing the recovery tasks (post‐SSM), main effects were found for sex in relative HF HRV during both sitting (*F*_(1, 46)_ = 7.81; *p* = 0.008; $\eta_{G}^{2}$ = 0.15 [moderate]) and standing (*F*_(1, 46)_ = 6.63; *p* = 0.013; $\eta_{G}^{2}$ = 0.14 [moderate]) (Figure 2). Relative LF HRV displayed a sex main effect during standing (*F*_(1, 46)_ = 6.63; *p* = 0.013; $\eta_{G}^{2}$ = 0.14 [moderate]) recovery but not sitting (*F*_(1, 46)_ = 3.05; *p* = 0.087; $\eta_{G}^{2}$ = 0.06 [small]) (Figure 2). The LF/HF ratio displayed sex main effects for both sitting (*F*_(1, 46)_ = 8.58; *p* = 0.005; $\eta_{G}^{2}$ = 0.16 [moderate]) and standing (*F*_(1, 46)_ = 12.2; *p* = 0.001; $\eta_{G}^{2}$ = 0.21 [moderate]) recovery (Figure 2). Lastly, pNN50 displayed sex main effects during standing recovery (*F*_(1, 46)_ = 5.34; *p* = 0.025; $\eta_{G}^{2}$ = 0.10 [small]) (Figure 1). No other main effects were found for sex (all *F*_(1, 46)_ < 3.51; all *p* > 0.067; all $\eta_{G}^{2}$ < 0.07 [negligible/small]), task (all *F*_(1, 46)_ < 2.77; all *p* > 0.103; all $\eta_{G}^{2}$ < 0.06 [negligible/small], or task by sex (all *F*_(1, 46)_ < 2.71; all *p* > 0.106; all $\eta_{G}^{2}$ < 0.06 [negligible/small]) (Figures 2 and 3).

### 3.6 HRV and BRS post hoc comparisons during recovery

Post hoc comparisons revealed females exhibited greater relative HF HRV activity in both seated (*p* = 0.008; Cohen's *d* = 0.81 [large]) and standing (*p* = 0.013; Cohen's *d* = 0.75 [moderate]) recovery (Figure 2). Females also exhibited less relative LF HRV activity during standing recovery (*p* = 0.013; Cohen's *d* = 0.75 [moderate]), and lower LF/HF ratios during both seated (*p* = 0.005; Cohen's *d* = 0.82 [large]) and standing (*p* = 0.001; Cohen's *d* = 0.99 [large]) recovery (Figure 2). Lastly, females displayed higher pNN50 (*p* = 0.025; Cohen's *d* = 0.67 [moderate]) during standing recovery (Figure 1).

## 4 DISCUSSION

The present study was the first investigation to measure sex differences in autonomic function both during and immediately following light resistance exercise through sustained repetitive BP oscillations (SSM). The main findings were: (1) despite eliciting larger increases in BP, no differences in autonomic function were found during or immediately following rSSM compared to bSSM, and (2) females displayed stronger vagal tone than males during recovery from both forms of SSM, as demonstrated through greater HF and lower LF values during quiet sitting and standing rest. Therefore, our findings suggest that a healthy ANS can manage the increased cardiovascular stressors accompanying light resistance exercise without requiring augmented recovery compared to bodyweight exercise. Our findings also suggest that females may experience ANS recovery more rapidly than males following light resistance exercise.

### 4.1 Comparisons with previous literature

To the authors’ knowledge, no study has sequentially captured ANS activity (HRV and BRS) both during resistance exercise capable of producing sinusoidal blood pressure oscillations as well as during the immediate recovery period (<10‐min). This is likely due to complexities surrounding participants being unable to perform heavy resistance exercise for a long enough duration to capture valid HRV and BRS estimates (Burma, Graver, et al., 2021). The present study used resistance intensities that were sustainable for the required 5‐min of HRV and BRS recording (bodyweight and 20% of bodyweight) such that ANS activity could be captured both during and immediately following resistance exercise.

Regarding general autonomic function, published studies have displayed that the intensity and duration of resistance exercise bouts acutely influence PNS activity and vagal tone recovery (Mayo, Iglesias‐Soler, Carballeira‐Fernández, et al., 2016; Mayo, Iglesias‐Soler, Fariñas‐Rodríguez, et al., 2016). Specifically, longer sets and working until volitional fatigue may apply greater stress to the ANS, blunting vagal recovery in healthy individuals (Mayo, Iglesias‐Soler, Carballeira‐Fernández, et al., 2016; Mayo, Iglesias‐Soler, Fariñas‐Rodríguez, et al., 2016). The present study examined resistance exercise intensity according to the amount of weight lifted (bodyweight vs. light resistance [20% of overall bodyweight]). It was found that, despite eliciting greater increases in BP, autonomic activity during and following rSSM was comparable to bSSM (Figures 1, 2, 3). As mentioned, this finding suggests a healthy ANS can respond appropriately to additional low‐level resistance (20% of bodyweight) without the need for augmented recovery compared to bodyweight exercise.

There is a paucity of literature delineating the effects of chromosomal sex on cardiovascular function, with the majority of cardiovascular research conducted on young, healthy males. This limits the generalizability and clinical utility of previous literature, given there are known chromosomal sex differences in ANS function (Koenig & Thayer, 2016). The research team is aware of only one study that examined sex‐specific differences in the ANS response to resistance exercise (Humm et al., 2021). Humm et al. (2021) examined HRV acutely following resistance exercise bouts, which comprised three sets of ten repetitions (at 70% of one‐repetition maximum) for five exercises (e.g., leg press, lateral pulldown, chest press, leg curl, and leg extension). Contrary to the findings of the present study, Humm et al. (2021) reported similar HRV recovery profiles across sex. Notably, the weight levels used for resistance exercise in Humm et al. (2021) (70% of one‐repetition maximum) were much higher than the present study (20% of bodyweight). Higher weight levels may have elicited greater BP increases and volitional fatigue, extensively stressing the ANS of participants. Extensively stressing participants’ ANS may have prevented quicker vagal recovery from being attainable (or measurable in a short timeframe) for females. In support of this, higher resistance exercise intensity has been linked to longer PNS recovery times (Mayo, Iglesias‐Soler, Carballeira‐Fernández, et al., 2016; Mayo, Iglesias‐Soler, Fariñas‐Rodríguez, et al., 2016).

The findings of the present study (stronger vagal tone displayed by females during recovery) parallel results from an investigation examining ANS activity post‐maximal aerobic exercise (Kappus et al., 2015). When comparing relative intensities (i.e., comparing maximal aerobic to maximal resistance exercise), aerobic exercise does not stress the ANS via BP elevations as much as resistance exercise (Davis et al., 2003; Di Francescomarino et al., 2009). Considered collectively, these findings suggest that healthy females may establish vagal tone faster than males following aerobic and light‐resistance exercise, but experience too great of a cardiovascular stressor (BP elevations) to do so following heavy resistance exercise. Therefore, the presence of some form of BP threshold may exist in which females can no longer autonomically recover quicker than males. Notably, baseline autonomic measures (HRV, BRS) were not collected in the present study, making it uncertain whether the discrepancy across sex in vagal tone following resistance exercise existed due to female's ability to autonomically recover from light resistance exercise quicker than males, or simply due to differences in autonomic levels at rest (Mendonca et al., 2010).

### 4.2 Physiological underpinnings—Cardiovascular and autonomic activity during and following exercise

To meet the elevated metabolic demands of exercise, a coordinated cardiovascular response occurs to augment cardiac output (Salmasi, 1993). This response involves decreased vascular afterload due to a drop in total peripheral resistance (Nobrega et al., 2014), increased end‐diastolic volume, and preload leading to amplified ventricle contraction via the Frank‐Starling mechanism (Kosta & Dauby, 2021). This increases stroke volume (Kosta & Dauby, 2021) and combines with elevations in heart rate (Salmasi, 1993) to augment the volume of blood pumped by the heart per minute. The ANS is majorly responsible for coordinating this response (Nobrega et al., 2014), as well as modulating fluctuations in BP throughout exercise. By doing so, the ANS drives increases in cardiac output while protecting regions vulnerable to pressure swings, such as peripheral vasculature and organs, most notably the brain (Smirl et al., 2018). After exercise, the ANS must recover from heightened activity by gradually re‐establishing vagal tone and decreasing sympathetic activity. The speed of recovery provides an index of autonomic health, such that poor PNS recovery and prolonged elevation in SNS output are associated with cardiac disease and poorer health outcomes (Goldsmith et al., 2000). Importantly, the level of cardiovascular and autonomic stress (i.e., BP elevations, increased myocardial activity) encountered during exercise influences the amount of required recovery (Mayo, Iglesias‐Soler, Carballeira‐Fernández, et al., 2016; Mayo, Iglesias‐Soler, Fariñas‐Rodríguez, et al., 2016). The findings of the present study suggest that cardiovascular stressors due to light resistance exercise via rSSM are not significant enough to require greater autonomic recovery than bodyweight exercise in healthy participants.

The physiological underpinnings surrounding the stronger vagal tone observed in females during recovery from resistance exercise may be due to differences in resting sex‐based autonomic activity. For example, at rest, healthy females display higher PNS sensitivity compared to males, and parallelly, males seem to be more sensitive to SNS activity, despite lower resting HR (Dart et al., 2002; Gupta & Kapoor, 2010; Ji et al., 2020; Joyner et al., 2016; Koenig & Thayer, 2016; Liao et al., 1995). There are a plethora of potential reasons for these baseline sex differences, however, most evidence supports a cardioprotective effect of estrogen leading to increased vagal tone in females (Dart et al., 2002; Du et al., 2006). As hormones were not measured in the current study, future research is warranted into the role of hormonal variation has on ANS recovery following exercise.

### 4.3 Clinical implications—Sport related concussion

The methodological approach of the present study may hold clinical utility by providing a technique in which ANS dysfunction could be quantified within clinical populations and if participants are able to perform resistance exercise safely. For example, within the realm of sport‐related concussion, there are exercise tests to understand if participants can safely return to aerobic exercise (Leddy & Willer, 2013). This is not the case with resistance exercise. Rather, the Concussion in Sport Group recommends starting resistance training at stage 4 of the graduated return‐to‐play recovery strategy (McCrory et al., 2017). However, as SRC is a heterogeneous injury (Collins et al., 2014; Kenzie et al., 2018), the timing for which resistance exercise can be reintroduced is likely to vary on a case‐by‐case basis. This likely stems from a dysregulation in cardiovascular and cerebrovascular function, where the massive BP oscillations are not adequately buffered (Wright et al., 2018). Conclusively, the specific methodological technique may therefore be considered a start to finding a more objective approach/comparison used to assess preparedness when returning to resistance exercise for the SRC population. Albeit more research is needed before the true clinical utility can be elucidated, especially given the methods used to quantify autonomic activity may not be readily available in clinical settings.

### 4.4 Limitations and future considerations

The present study is not without its limitations. Cardiovascular fitness and training status were not measured, which could have impacted the outcome variables of interest (i.e., HRV and BRS). Specifically, healthier and physically fit individuals may have exhibited lower HRV metrics which could have accounted for discovered sex differences during recovery. The populations utilized were young (average age of 23.2 ± 2.8 years) and seemingly healthy individuals capable of performing sustained SSM. Accordingly, the findings of this investigation may not be generalizable outside of young adults or to clinical populations. Furthermore, contraceptive use, menstrual cycle status, and sex hormone levels were not recorded, and therefore, the results should be taken in light of these considerations. This limits our ability to speculate that the hypothesized cardioprotective benefits of estrogen may be a factor in the stronger vagal recovery of females. Future research should investigate any correlation between hormone levels and ANS recovery from resistance exercise, as well as cardiovascular function in general. Notably, participants acted as their own controls when performing all four SSM activities. Henceforth, any modifying/confounding effect of hormones would only impact sex and task by sex comparisons, as all confounding factors would be accounted for within the task comparisons. The autonomic activity was not recorded at rest. This limits the present study's ability to infer whether discovered sex differences during recovery are exemplary of faster vagal recovery in females or simply representative of a cardioprotective disposition unique to females existing under resting conditions. Lastly, the methodology used by this study to assess ANS response to resistance exercise is not widely or clinically available, meaning the clinical suggestions put forth by this investigation are suggestive in nature. Future research is warranted to investigate the feasibility of integrating HRV and BRS analysis into the recovery program of clinical conditions with ANS dysfunction such as SRC.

## 5 CONCLUSION

The current study analyzed ANS activity (i.e., HRV, BRS) both during and following resistance exercise in a young, sex‐balanced, and healthy sample. Despite increased BP, no differences in HRV and BRS were discovered when increasing exercise intensity from bodyweight to light resistance (20% of bodyweight). This suggests a healthy autonomic system can attend to increases in BP accompanying light resistance without the need for augmented recovery compared to bodyweight work. Furthermore, a sex difference in autonomic response to resistance exercise appears to exist, as females displayed a stronger vagal tone than males during recovery. This suggests that females may exhibit faster PNS recovery post‐resistance exercise or that sex differences in HRV exist at rest in which females are more parasympathetically driven, both are cardioprotective traits. Lastly, the potential clinical application may exist by comparing the autonomic response to resistance exercise of healthy individuals with individuals from clinical populations with ANS dysfunction (e.g., SRC). Future research confirming the healthy reference of autonomic activity found within the present study, as well as comparing that to clinical populations such as SRC is required.

## CONFLICT OF INTEREST

The authors declare they have no conflicts of interest to report.

## AUTHOR CONTRIBUTION

Joseph Carere: Methodology, Investigation, Writing–Original draft, Writing–Review & Editing; Joel S. Burma: Conceptualization, Methodology, Formal analysis, Investigation, Writing–Original draft, Writing–Review & Editing, Visualization, Supervision; Kailey Newel: Conceptualization, Methodology, Investigation, Writing–Review & Editing; Courtney M. Kennedy: Conceptualization, Methodology, Writing–Review & Editing; Jonathan D. Smirl: Conceptualization, Resources, Writing–Review & Editing, Supervision, Funding acquisition.
